# Acute lymphoid and myeloid leukemia in a Brazilian Amazon population: Epidemiology and predictors of comorbidity and deaths

**DOI:** 10.1371/journal.pone.0221518

**Published:** 2019-08-22

**Authors:** Alexander Leonardo Silva-Junior, Fabíola Silva Alves, Marlon Wendell Athaydes Kerr, Lilyane Amorim Xabregas, Fábio Magalhães Gama, Maria Gabriela Almeida Rodrigues, Alexandre Santos Torres, Andréa Monteiro Tarragô, Vanderson Souza Sampaio, Maria Perpétuo Socorro Sampaio Carvalho, Nelson Abrahim Fraiji, Adriana Malheiro, Allyson Guimarães Costa

**Affiliations:** 1 Diretoria de Ensino e Pesquisa, Fundação Hospitalar de Hematologia e Hemoterapia do Amazonas (HEMOAM), Manaus, AM, Brazil; 2 Programa de Pós-Graduação em Ciências Aplicadas a Hematologia, Universidade do Estado do Amazonas (UEA), Manaus, AM, Brazil; 3 Programa de Pós-Graduação em Imunologia Básica e Aplicada, Instituto de Ciências Biológicas, Universidade Federal do Amazonas (UFAM), Manaus, AM, Brazil; 4 Programa de Pós-Graduação em Medicina Tropical, Universidade do Estado do Amazonas (UEA), Manaus, AM, Brazil; 5 Instituto de Pesquisa Clínica Carlos Borborema, Fundação de Medicina Tropical Dr. Heitor Vieira Dourado (FMT-HVD), Manaus, Amazonas, Brazil; 6 Fundação de Vigilância em Saúde do Amazonas, Manaus, Brazil; M D Anderson Cancer Center, UNITED STATES

## Abstract

**Introduction:**

Leukemia is the most common cancer in children and has the highest rates of incidence in industrialized countries, followed by developing countries. This epidemiologic profile can mainly be attributed to the availability of diagnostic resources. In Brazil, leukemia diagnosis is a challenge due to financial viability, lack of hemovigilance services in isolated regions and the vast size of the territory. Its incidence in the state of Amazonas has been increasing since 2010. Therefore, this study aims to describe the epidemiological pattern and spatial distribution of patients with acute lymphoid leukemia and acute myeloid leukemia in Amazonas and identify the predictors of comorbidity and death.

**Materials and methods:**

A retrospective cross-sectional study was carried out based on patients’ data which was obtained from the database of a referral center for the period of 2005 to 2015. Variables included age, gender, ethnicity, civil status, schooling, income, location of residence, subtype of leukemia, comorbidities, and date of death. The spatial distribution was performed using QGIS v.2.18. Stata software was used for univariable and multivariable logistic regression to evaluate the association between both comorbidities and death for all characteristic groups of ALL and AML.

**Results:**

The group that was studied was composed of 577 ALL and 266 AML patients. For both, most patients were male, with a schooling period of 1–4 years, received<1 minimum wage, and lived mostly in Manaus, followed by the municipality of Tefé. There was no association between the development of comorbidities and analyzed variables in patients with ALL. AML patients that were >60 years old and with family history of the disease had the highest risk of developing comorbidities (OR = 5.06, p = 0.038; OR = 2.44, p = 0.041, respectively). Furthermore, patients with ALL and in the 41-50-year age group had a higher risk of death (OR = 31.12; p = 0.001). No association between death and explanatory variables were found in patients with AML. In addition, significant difference was observed in time to death (chi2 = 4,098.32, p = 0.000), with 50% of patients with AML dying within two years after diagnosis, whereas in ALL, this percentual of death only is reached in approximately 5 years.

**Conclusion:**

Our study describes the data of patients with acute leukemia in Amazonas, a remote region in the north of Brazil. In addition, it highlights the importance of hemovigilance in an Amazon region state, while focusing on peripheral areas which don't have prevention, diagnosis and treatment tools for this disease.

## Introduction

Leukemia is a type of hematological neoplasm, characterized by disorders such as the proliferation of stem cells, which results in the loss of functional capacity of hematopoietic tissue [1]. Its etiology is not well elucidated; however, factors such as age, medical history, exposure to environmental factors, and presence of genetic mutations determine the level of risk and affect the clinical outcomes of this disease [2–5].

The diagnosis is based on morphological, immunophenotypic, karyotype, and/or molecular analysis, which leads to the classification of leukemia as either acute or chronic depending on the stage of maturation [6–8]. Acute Lymphoid Leukemia (ALL) is more frequent (predominance rate 75%) than Acute Myeloid Leukemia (AML) (predominance rate 25%), and is classified according to compromised cell lineage [2]. ALL has a greater level of incidence in patients of pediatric age (<15 years old), although there is a peak in incidence after 50 years of age [8–11]. In contrast, AML cases occur at a very high rate in adults (80%), with a higher frequency in people aged over 65 years [2,7,8,12,13].

The immunodeficiency state caused by the disease or induced by the chemotherapy, linked to a higher susceptibility to infections, makes this neoplasm the main cause of death in developed countries and the second most common form of death by disease in developing countries [6,10,11,14,15]. The world mortality rate for leukemia patients increased by 31,6% between 2005 and 2015, however, treatment protocols have been improved throughout the years with the development of new drugs, such as Inotuzumab [16,17].

Leukemia (acute and chronic) has the 11^th^ highest rate of incidence among all cancers worldwide and the number of cases is increasing over time, which leads to it being considered a public health issue [6,18–20]. Oceania, North America, and Europe are the regions with highest number of cases and frequency—40–50 cases per million habitants—especially in industrialized regions [21,22]. Developing countries register 1–40 new cases per million inhabitants, a low number that may be associated with misdiagnosis or unreported cases [22–24].

Epidemiological studies carried out between 1980 and 2004 in Latin America identified a higher incidence and mortality in Brazil, Colombia, and Mexico [20]. Brazilians have many risk factors for solid cancer and leukemia, such as ethnic and genetic diversity, exposure to chemicals and other carcinogenic substances; as well as the possibility of unreported cases since, as previously mentioned, misdiagnosis may influence the rate of incidence indifferent Brazilian regions [25,26].

The incidence of leukemia in Brazil has increased with time and currently represents the 9^th^ most common cancer. With a higher proportion in males, it causes from 41.7 to 57.5 cases per million inhabitants [23,27]. Brazilian national records demonstrate that this cancer occurred in 180.988 people in 2011, with a mortality rate of 16,4%, and is thus the third most common cause of death among the non-transmissible chronic diseases [28]. The proportion of leukemia cases among regions is variable and has increased since the last report by the Instituto Nacional do Câncer (INCA) [23,27,29,30].The Northern region was identified as the region with the second highest number of leukemia cases and is one of the regions with the lowest municipal human development index (mHDI) and highest childhood mortality rate [24,31,32].

Amazonas has the greatest territorial extension of all the federal units and its capital, Manaus, was identified in 2010 as the capital with the second highest age adjusted incidence rate (under 14) of leukemia in children and adolescents [9]. In 2011, it had the highest rate of incidence in the country, with 76,8 leukemia cases per million inhabitants [26], which further increased in 2016 to 87,1 [33]. However, there is only one center for diagnosis and treatment of leukemia in the State (Fundação Hospitalar de Hematologia e Hemoterapia do Amazonas [HEMOAM]); and this center faces challenges in its hematological vigilance of the region due to the vast size of the territory and since most inter-municipal access is via waterways.

In order to describe the epidemiological profile of patients diagnosed with ALL and AML in the Amazon region, this study evaluated the spatial distribution of the disease, municipalities with higher numbers of cases, epidemiological and clinical patterns, and risk factors of acute leukemia in Amazonas. Our study shows a new analysis of acute leukemia patients and is the first to describe the epidemiological pattern in Amazonas by focusing on geographical distribution and factors that predict comorbidity and death.

## Materials and methods

### Study area

According to the Brazilian Institute of Geography and Statistics (IBGE), Amazonas has a geographic distribution of 1.559.146,876 km^2^, divided into 62 municipalities. Manaus, the capital of the State, is located in the east of the state. With a territorial area of 11.401,092 km^2^, Manaus has the highest demographic density (158,06hab/km^2^), and the highest municipal HDI of the state [34]. HEMOAM is located in Manaus and is the state referral center for the diagnosis, treatment and monitoring of patients with hematological diseases, especially leukemia, anemia, hemoglobinopathies, hemophilia, and thalassemia. It treats all cases in the Amazon territory and nearby municipalities which belong to other federal states.

### Ethics statement

This study was approved by the Ethical Review Committee at Fundação Hospitalar de Hematologia e Hemoterapia do Amazonas (CEP-HEMOAM), under protocol number 1.624.101/2016. The institutional review board waived the need for written informed consent from participants as the study involved secondary data and the confidentiality of patients’ identities was protected.

### Data source

This study was conducted using a retrospective cross-sectional approach. Each patient’s data was collected from the Statistical and Medical Record System (SAME), Cancer Hospital Registry (RHC), and the physical data file. We included patients with confirmed diagnosis of both lymphoid and myeloid acute leukemia between 2005 and 2015 at HEMOAM.

Demographic data was classified into the following categories: age (intervals of 10 years up to 60 years of age); gender; ethnicity (white, admixed, black, indigenous or yellow) as per database record; civil status (single, married, divorced, widow or consensual union); schooling; income (divided into units of Brazilian minimum wages per month [1 minimum wage was R$ 954,00 in 2018]); location of residence; family records (qualitative presence of genetic disorders or other non-infectious diseases in older family members); and date of death (those who died before the end of study). Clinical data of ALL subtype was classified into subtypes B, T, or mixed (for those with B and T leukemia), following the WHO recommendation, while the AML patients were divided according to French-American-British (FAB) classification system recommendation in M0-M7 [35,36].The comorbidities included hepatomegaly, splenomegaly, hepatosplenomegaly, adenomegaly, previous infectious diseases with IgG+, systemic arterial hypertension (SAH) and others (genetic disorders, hypertrophy, previous neoplasms, transfusions, metabolic disorders, etc).

### Descriptive and statistical analysis

Spatial distribution of the ALL and AML cases were performed using QGIS v.2.18.4 software (QGIS Development Team, QGIS Geographic Information System, and Open Source Geospatial Foundation Project). Data and its completeness are presented in the tables. Univariate and multivariate logistic regressions were performed in order to investigate associations between the comorbidities and death in ALL and AML. For both regression analyses, the variables for age, gender, ethnicity, civil status, schooling, income, place of residence and family history were included as confounders. A backward stepwise technique was applied. Variables with p-values ≤0.2 in the simple regression were selected for the multiple model analysis. Kaplan-Meier method was used for survival analysis that demonstrated the time to death in 10 years after diagnosis for ALL and AML patients. Statistical analysis was performed with log-rank test for comparison between groups. The final model considered all variables that were statistically significant (p<0.05). Statistical analysis was performed using package STATA v.13 (StataCorp, 2013, College Station, Texas, USA).

## Results

**Fig 1:**
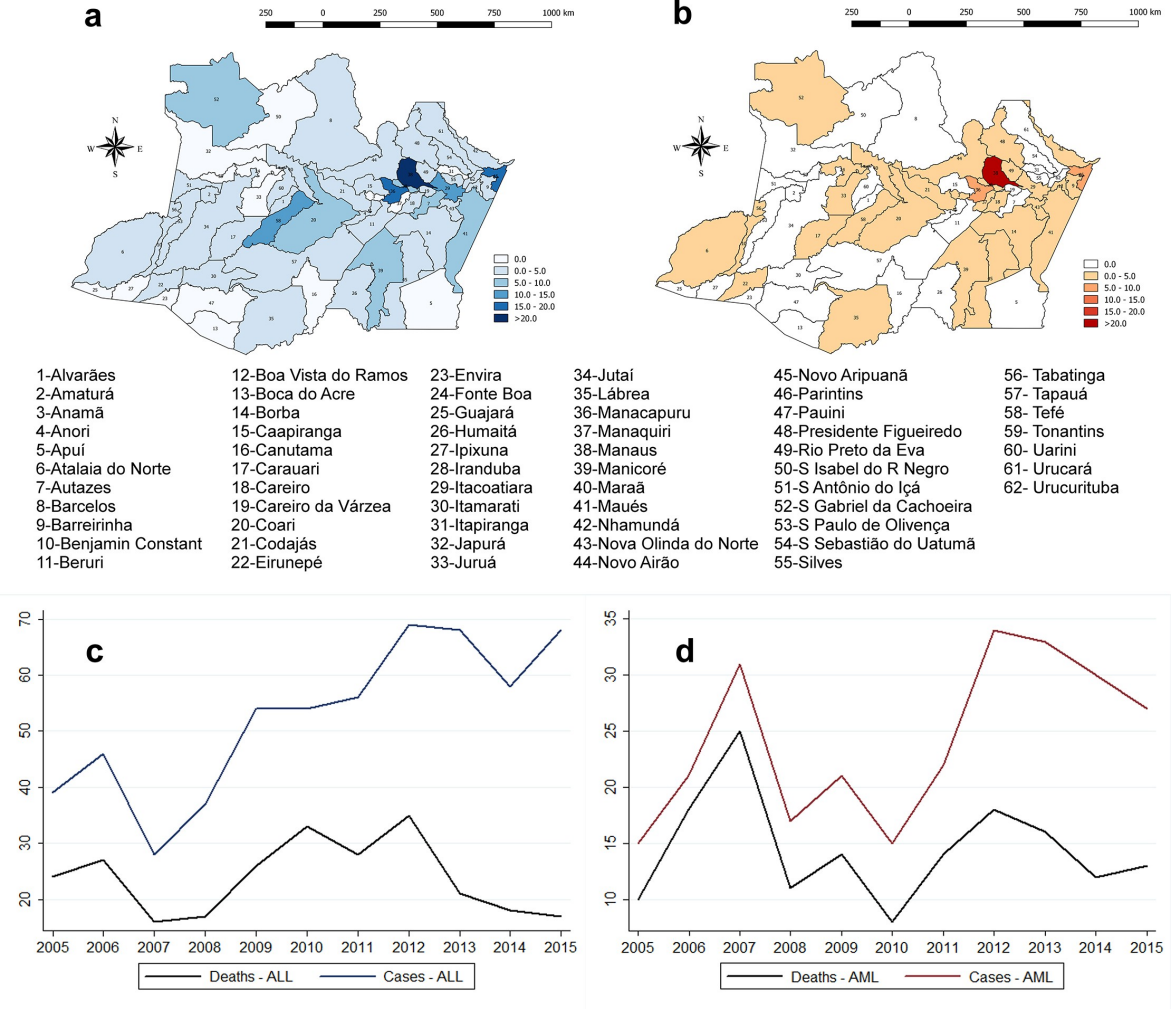
Spatial distribution by municipality in Amazonas and number of cases and death per year of acute leukemia patients diagnosed between 2005 and 2015. a) ALL patient distribution. b) AML patient distribution. c) Number of cases and deaths of ALL patients per year of study. d) Number of cases and deaths of AML patients per year of study.

A total of 843 (ALL, 577 [68.45%] and AML, 266 [31.55%]) acute leukemia patients were diagnosed at HEMOAM (proportion of 2.16 ALL cases to AML cases) between 2005 and 2015 (Fig 1A and 1B). The highest number of cases (ALL, 69 and AML, 34) were registered in 2012 (Fig 1). Of the ALL and AML patients, 558 (96.70%) and 253 (95.11%), respectively, had their place of residence registered. Most of the patients lived in Manaus (310 [55.56%] ALL; 165 [65.22%] AML). Acute leukemia cases were also recorded in other municipalities of Amazonas, such as Manacapuru and Parintins. The interior municipalities with higher frequency of ALL were Tefé and Maués (11 and 10 cases, respectively), while the other municipalities had less than 10 cases each. Regarding AML, Tefé had the highest number of cases (8 cases per 100,000 inhabitants), while the others had <6 cases. There were no registered cases of acute leukemia in the municipalities of Apuí, Boca do Acre, Canutama, Envira, Guajará, Ipixuna, Itapiranga, Japurá, Juruá, Pauini, Santa Isabel do Rio Negro and Tonantins, representing almost 20% of the Amazonas State population. Also, less than 10% of the patients diagnosed with acute leukemia declared residence in other states.

Regarding the ALL cases, 459 (79.55%) were diagnosed with subtype B, 52 (9.01%) with T, and only one case was registered as biphenotypic(T/B). The main subtype of the AML patients was M3 (13.16%), followed by M5 (10.90%), M2 (10.53%), M1 (6.77%), M7 and M4 (3.76% each), M0 (2.63%), and M6 (0.75%). The number of patients without assigned subtypes were 65/577 (11.27%) for ALL and 129/266 (48.50%) for AML.

The ALL patients were mostly in the age group ≤10 years (325 cases), followed by 122 patients aged 11–20 years, with all other age groups accounting for <10% of cases. Among AML patients, the age of 261 of 266 patients was available. The largest single group of patients was aged >60 years old (26%), followed by those between 11–20 years old (16%) and the other groups corresponded to <15% in our study (Table 1).

**Table 1:** Epidemiological characteristics of patients diagnosed with ALL and AML in the Amazonas State.

Characteristic (Completeness)	ALL n (%)	AML n (%)
**Age**	**572 (99.13)**	**261 (98.12)**
0–10	325 (56.82)	37 (14.18)
11–20	122 (21.33)	44 (16.86)
21–30	39 (6.82)	31 (11.88)
31–40	24 (4.19)	23 (8.81)
41–50	23 (4.02)	30 (11.49
51–60	18 (3.15)	28 (10.73)
> 60	21 (3.67)	68 (26.05)
**Gender**	**577 (100)**	**266 (100)**
Male	328 (56.85)	151 (56.77)
Female	249 (43.15)	115 (43.23)
**Ethnicity**	**507 (87.86)**	**230 (86.46)**
White	99 (19.52)	55 (23.91)
Admixed	384 (75.74)	165 (71.74)
Black	9 (1.78)	6 (2.61)
Indian	11 (2.17)	2 (0.87)
Yellow	4 (0.79)	2 (0.87)
**Civil status**	**537 (93.06)**	**232 (87.2)**
Single	465 (86.59)	116 (50.00)
Married	47 (8.75)	78 (33.62)
Divorced	2 (0.37)	13 (5.60)
Widow	3 (0.56)	14 (6.04)
Consensual union	20 (3.73)	11 (4.74)
**Schooling**	**332 (57.53)**	**115 (43.23)**
None	69 (20.78)	9 (7.83)
1–4 years	209 (62.95)	46 (40.0)
5–8 years	33 (9.94)	23(20.0)
>8 years	21 (6.33)	37 (32.17)
Income*	**381 (66)**	**140 (52.6)**
0–1 minimum wage	205 (53.81)	65 (46.43)
2–3 minimum wages	145 (38.06)	58 (41.43)
>3 minimum wages	31 (8.13)	17 (12.14)
**Residence**	**558 (96.70)**	**253 (95.11)**
Manaus	310 (55.56)	165 (65.22)
Interior of Amazonas	196 (35.12)	66 (26.09)
Other states	52 (9.32)	22 (8.69)
**Family history**	**172 (29.80)**	**123 (46.24)**
Yes	85 (49.42)	71 (57.72)
No	87 (50.58)	52 (42.28)
**Comorbidity**	**577 (100)**	**266 (100)**
Yes	348 (60.31)	181 (68.05)
No	229 (39.69)	85 (31.95)
**Death**	**577 (100)**	**266 (100)**
Yes	262 (45.41)	159 (59.77)
No	315 (54.59)	107 (40.23)

The highest number of cases occurred in men, with 328 and 151 cases for ALL and AML, respectively (see Table 1). Although more frequent in men, higher rates were observed for women in 2008 for ALL, and in 2005, 2006, 2010, and 2011 for AML. Approximately, 50% of our cases for both acute leukemia types were of a marital status classified as single, followed by married, while other types of civil status showed less than 10%, as demonstrated in Table 1.

Even though only 332 (57.53%) ALL and 115 (43.23%) AML patients had data available regarding the amount of schooling received, most acute leukemia patients had 1–4 years of schooling. Among ALL patients, 69 had received no schooling and for AML, 37 patients had had ≥8 years of schooling and 23 had had 5–8 years. The other groups did not show no percentage higher than 10% for both types of leukemia (Table 1). Most of the acute leukemia patients received ≤1 minimum wage per month (205 for ALL and 65 for AML). These numbers were followed by the group who received less than two and three minimum wages (145 and 58, respectively).

Although 172 (29.80%) of ALL and 123 (46.24%) of AML patients had their family history leukemia registered, we identified that 85 (49.42%) and 71 (57.72%) patients, respectively, had no family record (Table 1). Of the 577 ALL patients, 348 had some form of comorbidity at diagnosis, with the most frequent being previous infectious diseases (n = 175 [38.55%]), followed by Systemic Arterial Hypertension (SAH) (n = 15 [3.30%]); and of the 266 AML patients, 181 (68.80%) had comorbidities, with the most common also being previous infectious diseases (93 [32.51%]), followed by SAH (25 [8.74%]), as shown in Table 2.

**Table 2:** Major comorbidities in patients diagnosed with ALL and AML.

Comorbidities	ALL n = 454	AML n = 286
IgG+ Infectious diseases, n (%)	175 (38.55)	93 (32.51)
SAH, n (%)	15 (3.3)	25 (8.74)
DM, n (%)	6 (1.32)	12 (4.2)
Down syndrome, n (%)	10 (2.20)	0
Aplasia, n (%)	12 (2.64)	10 (3.5)
Others, n (%)	236 (51.99)	146 (51,05)

IgG: Immunoglobulin G; SAH: Systemic Arterial Hypertension; DM: Diabetes mellitus

There was no association between the development of comorbidities and any age groups, gender, ethnicity, civil status, school, income, residence, family records, or death for the patients with ALL (Table 3). Patients with AML ≥60 years old showed a 5-fold risk of developing comorbidities (OR = 5.06; p = 0.038). Also, we found that those who had family records had almost a three-fold risk of developing a comorbidity (OR = 2.44; p = 0.041).

**Table 3:** Logistic regression with univariable and multivariable of comorbidities and epidemiological characteristics in ALL and AML patients.

Variables	Comorbidities		ALL				Comorbidities		AML			
	Yes (%)	No (%)	Crude OR (CI 95%)	p value	Adjusted OR (CI 95%)	p value	Yes (%)	No (%)	Crude OR (CI 95%)	p value	Adjusted OR (CI 95%)	p value
**Age**												
**0–10**	183 (53.20)	142 (62.28)	-	1	-	1	21 (11.73)	16 (19.51)	-	1	-	1
11–20	74 (21.51)	48 (21.05)	0.96 (0.63–1.47)	0.868	-	1	30 (16.77)	14 (17.08)	1.03 (0.41–2.59)	0.943	-	1
21–30	25 (7.27)	14 (6.14)	1.29 (0.64–2.57)	0.467	-	1	19 (10.61)	12 (14.63)	0.93 (0.34–2.53)	0.896	-	1
31–40	16 (4.65)	8 (3.5)	1.20 (0.51–2.83)	0.668	-	1	15 (8.39)	8 (9.75)	1.35 (0.43–4.14)	0.600	-	1
41–50	17 (4.94)	6 (2.64)	1.35 (0.55–3.28)	0.500	-	1	20 (11.17)	10 (12.20)	1.02 (0.37–2.80)	0.968	-	1
51–60	12 (3.49)	6 (2.64)	2.53 (0.81–7.86)	0.108	2.88 (0.61–13.60)	0.180	19 (10.61)	9 (10.98)	1.47 (0.50–4.30)	0.474	-	1
> 60	17 (4.94)	4 (1.75)	2.31 (0.82–6.47)	0.110	-	1	55 (30.72)	13 (15.85)	***2*.*23*** ***(0*.*90–5*.*52)***	***0*.*081***	***5*.*06*** ***(1*.*09–23*.*48)***	***0*.*038***
**Gender**												
Male	189 (54.31)	139 (60.7)	-	1	-	1	106 (58.56)	45 (52.9)	-	1	-	1
Female	159 (45.69)	90 (39.3)	0.94 (0.67–1.33)	0.763	-	1	75 (41.44)	40 (47.1)	1.02 (0.61–1.73)	0.913	-	1
**Ethnicity**												
White	53 (17.2)	46 (23.11)	-	1	-	1	41 (25.95)	14 (19.44)	-	1	-	1
Admixed	243 (79.0)	141 (70.86)	1.45 (0.92–2.28)	0.186	1.18 (0.77–1.82)	0.432	114 (72.15)	51 (70.83)	0.73 (0.36–1.46)	0.383	-	1
Black	6 (1.95)	3 (1.5)	1.47 (0.34–6.23)	0.523	-	1	2 (1.26)	4 (5.56)	0.17 (0.02–1.03)	0.055	0.61 (0.04–7.55)	0.704
Indian	3 (0.97)	8 (4.03)	0.27 (0.06–1.10)	0.949	-	1	1 (0.64)	1 (1.39)	0.34 (0.02–5.82)	0.458	-	1
Yellow	3 (0.97)	1 (0.5)	2.21 (0.22–22.00)	0.270	-	1	0	2 (2.78)	-	1	-	1
**Civil Status**												
Single	279 (84.54)	186 (89.85)	-	1	-	1	72 (46.15)	39 (54.93)	-	1	-	1
Married	34 (10.3)	13 (6.28)	1.07 (0.58–1.99)	0.811	-	1	53 (33.97)	25 (35.21)	1.15 (0.62–2.13)	0.656	-	1
Divorced	2 (0.61)	0	-	1	-	1	10 (6.41)	3 (4.22)	2.98 (0.62–14.17)	0.169	0.86 (0.66–11.31)	0.913
Widow	2 (0.61)	1 (0.49)	-	1	-	1	12 (7.7)	2 (2.82)	1.35 (0.39–4.61)	0.625	-	1
Consensual unions	13 (3.94)	7 (3.38)	2.67 (0.88–8.13)	0.083	2.19 (0.70–6.79)	0.174	9 (5.77)	2 (2.82)	2.44 (0.50–11.88)	0.269	-	1
**Schooling**												
None	32 (16.58)	37 (26.62)	-	1	-	1	5 (7.05)	4 (9.1)	-	1	-	1
1–4	124 (64.25)	85 (61.15)	1.40 (0.96–2.88)	0.228	-	1	27 (38.02)	19 (43.18)	0.77 (0.17–3.51)	0.744	-	1
5–8	20 (10.36)	13 (9.35)	1.35 (0.64–3.47)	0.475	-	1	15 (21.13)	8 (18.18)	0.93 (0.18–4.78)	0.938	-	1
>8	17 (8.81)	4 (2.88)	***4*.*24*** ***(1*.*25–13*.*53)***	***0*.*017***	1.05 (0.46–2.38)	0.892	24 (33.8)	13 (29.54)	0.92 (0.19–4.31)	0.919	-	1
**Family history**												
No	59 (48.36)	28 (56)	-	1	-	1	32 (34.78)	21 (64.52)	-	1	-	1
Yes	63 (51.64)	22 (44)	0.96 (0.51–1.81)	0.913	-	1	60 (65.22)	11 (35.48)	***2*.*74*** ***(1*.*20–6*.*23)***	***0*.*016***	***2*.*44*** ***(1*.*03–5*.*76)***	***0*.*041***
**Death**												
No	176 (50.57)	139 (60.7)	-	1	-	1	62 (34.25)	45 (52.94)	-	1	-	1
Yes	172 (49.43)	90 (39.3)	1.13 (0.81–1.59)	0.451	-	1	119 (65.75)	40 (47.06)	***1*.*71*** ***(1*.*01–2*.*89)***	***0*.*045***	2.20 (0.92–5.26)	0.075

Out of the 843 patients, 421 (49.94%) died between the diagnosis and the end of 2016 (262 [45.41%] ALL and 159 [59.77%] AML) (Table 1). Although the rate of new ALL cases increased from 2011, the death rate decreased as of 2012 (Fig 1C). On the other hand, AML patients showed higher mortality rates until 2011 (Fig 1D).

**Fig 2:**
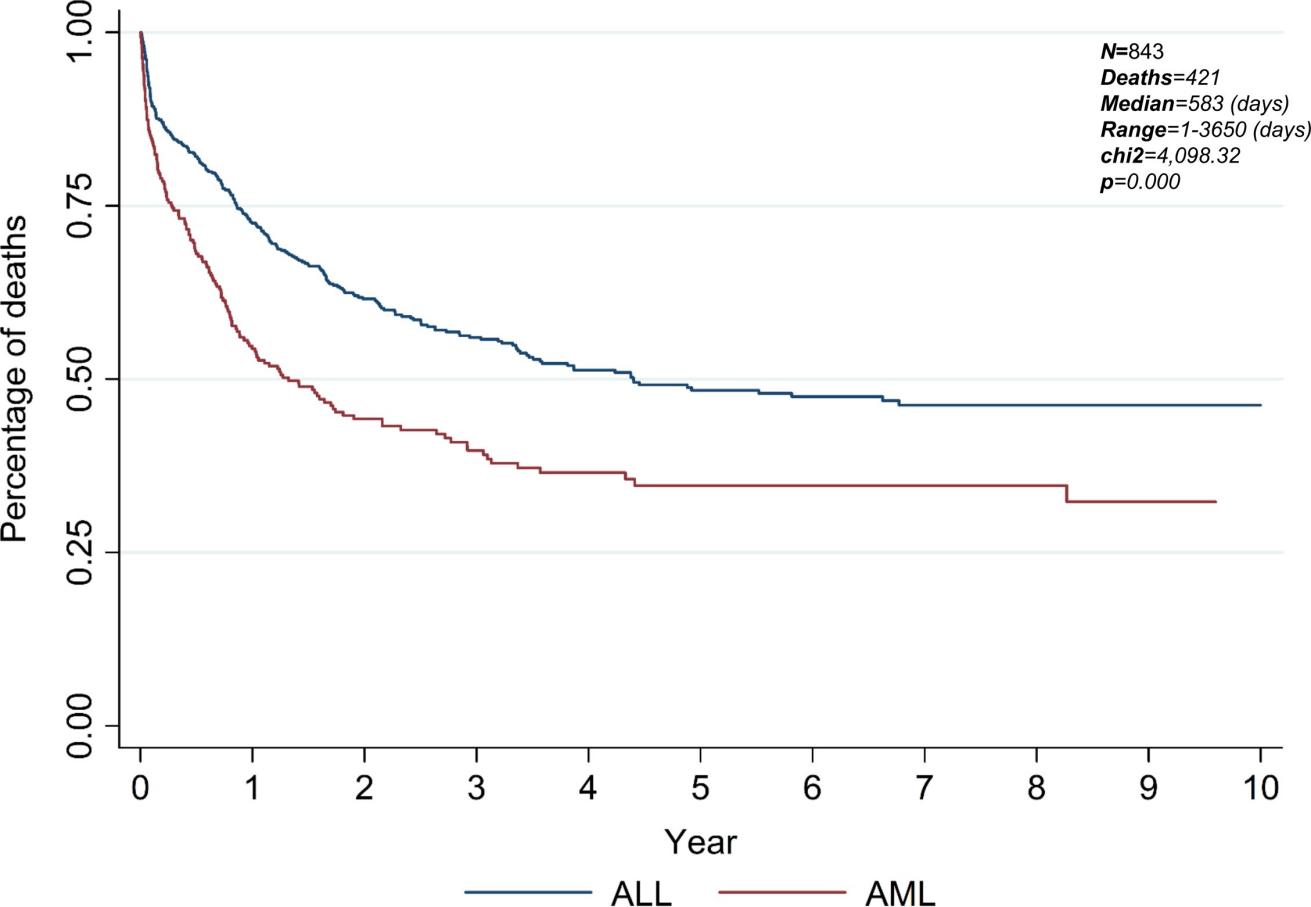
Time to death in 10 years after diagnosis for both types of acute leukemia patients (ALL and AML). Kaplan-Meier method was used for survival analysis demonstrating that the time to patient death in the AML was different from that of the ALL (N = 843, Deaths = 421, Median = 583 (days), Range = 1–3650 (days), chi^2^ = 4,098.32, p = 0.000). Furthermore, statistical analysis was performed with log-rank test for comparison between groups.

Patients with ALL and in the 41–50 year age group have a 31-fold risk of death (OR = 31.12; p = 0.001), while those in the 11–20 year group show more than a two-fold risk (OR = 2.22; p = 0.000), and the 21–30 and 51–60 groups have a 4-fold risk of death (OR = 4.89; p = 0.000; OR = 4.89; p = 0.008, respectively), as shown in Table 4. All other parameters of ALL showed no statistical significance for adjusted analyses. No association between death and explanatory variables were found in patients with AML. In addition, the percentage of death was higher in AML patients compared to their ALL counterparts (Fig 2). Significant difference was observed in time to death (chi^2^ = 4,098.32, p = 0.000), with 50% of patients with AML dying within two years after diagnosis, whereas in ALL, this number is reached in approximately 5 years.

**Table 4:** Logistic regression with univariable and multivariable of death and epidemiological and clinical characteristics in ALL and AML patients.

Variables	Death		ALL				Death		AML			
	Yes (%)	No (%)	Crude OR (CI 95%)	p value	Adjusted OR (CI 95%)	p value	Yes (%)	No (%)	Crude OR (CI 95%)	p value	Adjusted OR (CI 95%)	p value
**Age**												
0–10	107 (41.31)	218 (69.65)	-	1	-	1	20 (12.82)	17 (16.19)	-	1	-	1
11–20	65 (25.1)	57 (18.21)	***2*.*32*** ***(1*.*52–3*.*55)***	***0*.*000***	***2*.*22*** ***(1*.*42–3*.*48)***	***0*.*000***	23 (14.74)	21 (20)	0.93 (0.38–2.23)	0.873	-	1
21–30	28 (10.81)	11 (3.51)	***5*.*18*** ***(2*.*48–10*.*81)***	***0*.*000***	***4*.*89*** ***(2*.*26–10*.*61)***	***0*.*000***	19 (12.18)	12 (11.43)	1.34 (0.51–3.54)	0.548	-	1
31–40	14 (5.4)	10 (3.2)	***2*.*85*** ***(1*.*22–6*.*63)***	***0*.*015***	0.93 (0.07–11.78)	0.958	15 (9.61)	8 (7.62)	1.59 (0.54–4.66)	0.395	-	1
41–50	20 (7.72)	3 (0.96)	***13*.*58*** ***(3*.*94–46*.*71)***	***0*.*000***	***31*.*12*** ***(4*.*08–237*.*26)***	***0*.*001***	16 (10.26)	14 (13.33)	0.97 (0.36–2.55)	0.953	-	1
51–60	13 (5.02)	5 (1.6)	***5*.*29*** ***(1*.*84–15*.*24)***	***0*.*002***	***4*.*89*** ***(1*.*51–15*.*77)***	***0*.*008***	17 (10.9)	11 (10.48)	1.31 (0.48–3.55)	0.592	-	1
> 60	12 (4.64)	9 (2.87)	***2*.*71*** ***(1*.*11–6*.*64)***	***0*.*029***	1.46 (0.08–26.11)	0.796	46 (29.49)	22 (20.95)	1.77 (0.78–4.04)	0.170	-	1
**Gender**												
Male	144 (54.96)	184 (58.41)	-	1	-	1	88 (55.35)	63 (58.88)	-	1	-	1
Female	118 (45.04)	131 (41.59)	1.15 (0.82–1.60)	0.405	-	1	71 (44.65)	44 (41.12)	1.15 (0.70–1.89)	0.569	-	1
**Ethnicity**												
White	52 (22.71)	47 (16.90)	-	1	-	1	40 (28.37)	15 (16.86)	-	1	-	1
Admixed	166 (72.49)	218 (78.42)	0.68 (0.44–1.07)	0.098	0.66 (0.43–1.01)	0.060	95 (67.37)	70 (78.65)	***0*.*50*** ***(0*.*26–0*.*99)***	***0*.*048***	-	1
Black	3 (1.31)	6 (2.16)	0.45 (0.10–1.90)	0.280	-	1	3 (2.13)	3 (3.37)	0.37 (0.06–2.06)	0.260	-	1
Indian	6 (2.62)	5 (1.8)	1.08 (0.31–3.78)	0.899	-	1	2 (1.42)	0	-	1	-	1
Yellow	2 (0.87)	2 (0.72)	0.90 (0.12–6.67)	0.921	-	1	1 (0.71)	1 (1.12)	0.37 (0.02–6.38)	0.498	-	1
**Civil status**												
Single	183 (76.25)	282 (94.95)	-	1	-	1	62 (46.97)	49 (51.58)	-	1	-	1
Married	35 (14.59)	12 (4.04)	***4*.*49*** ***(2*.*27–8*.*88)***	***0*.*000***	***1*.*39*** ***(0*.*37–5*.*14)***	0.620	45 (34.09)	33 (34.74)	1.07 (0.60–1.93)	0.802	-	1
Divorced	2 (0.83)	0	-	1	-	1	7 (5.30)	6 (6.32)	0.92 (0.29–2.92)	0.890	-	1
Widow	2 (0.83)	1 (0.34)	3.08 (0.27–34.23)	0.360	-	1	12 (9.09)	2 (2.10)	***4*.*74*** ***(1*.*01–22*.*18)***	***0*.*048***	-	1
Consensual union	18 (7.5)	2 (0.67)	***13*.*86*** ***3*.*18–60*.*48)***	***0*.*000***	-	1	6 (4.55)	5 (5.26)	0.94 (0.27–3.29)	0.933	-	1
**Schooling**												
None	21 (13.04)	48 (28.07)	-	1	-	1	4 (5)	5 (14.29)	-	1	-	1
1–4	104 (64.6)	105 (61.40)	***2*.*26*** ***(1*.*26–4*.*04)***	***0*.*006***	0.98 (0.30–3.16)	0.974	33 (41.25)	13 (37.14)	3.17 (0.73–13.70)	0.122	0.85 (0.07–9.06)	0.893
5–8	20 (12.42)	13 (7.60)	***3*.*51*** ***(1*.*47–8*,*36)***	***0*.*004***	0.76 (0.29–1.96)	0.580	16 (20)	7 (20)	2.85 (0.58–13.96)	0.195	3.20 (0.58–17.66)	0.181
>8	16 (9.94)	5 (2.93)	***7*.*31*** ***(2*.*36–22*.*58)***	***0*.*001***	2.02 (0.62–6.57)	0.239	27 (33.75)	10 (28.57)	3.37 (0.75–15.15)	0.112	0.72 (0.17–2.89)	0.645
**Family history**												
No	37 (44.58)	50 (56.18)	-	1	-	1	31 (36.9)	21 (53.85)	-	1	-	1
Yes	46 (55.42)	39 (43.82)	1.59 (0.87–2.91)	0.129	1.24 (0.51–3.01)	0.631	53 (63.1)	18 (46.15)	1.99 (0.92–4.30)	0.079	2.28 (0.67–7.80)	0.185

## Discussion

In Brazil, leukemia is a public health issue in many regions, especially because of scarce financial support for the diagnosis in the states [25]. Since Amazonas has the largest territorial extension of all states, HEMOAM faces a challenge related to hemovigilance at the municipal level.

In this study, we identified 843 cases of acute leukemia which were diagnosed at HEMOAM during a 10 year-period (2005–2015), with an average of 84 new a cases per year. As expected, this rate is lower than the values described for developed countries, such as Canada [37] and USA [8], which leads us to reason that the identification methods of new cases are not performed by professionals from municipalities. Those professionals are responsible for identifying the suspected leukemia cases by microscopy, and then refer them to HEMOAM for the correct diagnosis and treatment.

In 2010 and 2012, we identified 69 and 103 acute leukemia cases, respectively, these numbers were lower than estimated by INCA [38,39]. However, estimates presented in the INCA report are based on the general amount of leukemia patients (which includes those of chronic leukemia), while our quantitative results are based on patients diagnosed with acute leukemia, which suggests that the incidence of acute leukemia in Amazonas is underestimated. This incidence, which is not very well documented, is due to the financial and infrastructural challenges of diagnostic centers, the patients’ difficulty in reaching referral centers, and the delay between the onset of symptoms and the correct diagnosis with subsequent effective treatment [9,11,22].

A higher number of ALL, in the age group of 0–10 years and B subtype is shown here and corroborates with other studies which describe it as the most common epidemiological pattern, however, the etiological factor was not analyzed in our study [10,11,15,22,25,40,41].

AML was more common in adults aged ≥60 years, with a 5-fold risk of developing some comorbidity, followed by the age group <20 years (30%), which suggests a higher susceptibility to comorbidity development. Since the main subtype of AML was the promyelocytic cell type (M3), which is an important progenitor from myeloid cells, with no differentiation and limited functionality, this may be a contributing factor to a worse prognosis [10,32,42].

Comparing results presented here to those of Junior *et al*., in a study that was carried out in the Pará State, they observed a higher number of cases of the AML M2 subtype followed by the M0/M1, where both had no differentiated lineage, such as M3, which is shown by our results [13].

Our results show a higher general frequency of both types of leukemia in male patients, however, Oliveira *et al*. (2009) reported that Porto Alegre and Belém registered more ALL and AML cases in females than in males of pediatric age [25]. Barbosa *et al*. studied the ALL patients in Belém and confirmed the same majority of cases involving females [11].

Since a higher frequency of leukemia in the admixed ethnicity with a two-fold chance of developing comorbidity has been shown, the hypothesis that miscegenation has an influence on the disease must be considered [10,43].Studies conducted in other states of Brazil, such as Santa Catarina [10], and other countries, such as Canada [44] and the United States of America [8] found more leukemia cases in white people. However, there might have been a bias, since the data regarding race was collected based on self-reporting by the patients.

Most of the ALL patients of the interior of the state had had 1–4 years of schooling and received <1 minimum wage, probably because rural labor is the predominant economic activity in the municipalities of the interior, which increases the exposition to environmental factors related to leukemia development, such as pathogens (Epstein-Barr Virus and Cytomegalovirus) and myelotoxic agents [11,13,22].

Manaus has already been indicated as being the capital with the highest number of leukemia cases in Brazil [22,26]. Most patients of both acute leukemia types (ALL and AML) reside in Manaus, which leads us to believe that Manaus’s greater financial resources allows for a better diagnosis and better reporting when compared to the other municipalities of Amazonas [15,41,45]; industrialization and exposition to tobacco, environmental pollution, and other risk factors related to cancer development have a tendency to increase incidence rates in the capital [11,46]; unreported cases occur in the municipalities of the interior due to the low number of professionals that are specialized in this type of health-care [10,26] and the great distance between the municipalities of the interior and an effective diagnostic center [41]. The last hypothesis is sustained by the fact that most cases are in municipalities close to Manaus.

More AML patients declared a previous family record of hereditary disease with a two-fold risk of developing comorbidities. To our knowledge, this data has not been discussed in the literature regarding patients who developed leukemia, but since the etiology of this neoplasm is not known, we suggest more studies regarding the families’ medical records and their genetic role in AML susceptibility in order to be able to conclude whether it has some influence on the onset of leukemia.

More than 60% of both types patients with acute leukemia had comorbidities at diagnosis. Even though our result does not describe the cause of their death, many studies portray the impact of different pathogens such as influenza, respiratory infections, hepatitis C virus, and human T-cell lymphotropic virus (HTLV) 1, on a worse prognosis [40,47,48].

The death rate for both ALL and AML patients shown here allows us to conclude that the improvement of therapy protocols, such as diagnostic methods, have valuable effects as could be evidenced by the lower percentage of deaths 10 years later. Almost half of the patients died before the end of our study. Our analysis showed that people with ALL in the 41 and 50-year-old age group have a 31-fold risk of death. This fact was associated with the immune instability and risk factors, suggesting a cause for the high number of deaths in this age group.

However, we must consider the following limitations to our study, such as:(i) residence information was obtained by patients’ report, and since patients from the interior may have consultations in Manaus which may involve a temporary stay in the city, they may cite their residence as being in Manaus; (ii) the diagnostic method chosen by the municipalities, due to financial issues, was morphological analysis, which makes it difficult to identify new leukemia cases and then get these patients transported to HEMOAM, especially in the case of municipalities that are a great distance from the capital; (iii) leukemia has no characteristic symptom, and many patients are misdiagnosed before they reach HEMOAM for the diagnosis and improved treatment, which raises the mortality rate and finally (iv) some patients who did not have enough data were excluded from this study. In addition, the logistic regression models used have some limitations in relation to the described comorbidity and death analyses.

## Conclusion

This study is the first to describe the epidemiological pattern using an active data search, which provided a better picture of the data of patients with acute leukemia in Amazonas. We highlight the importance of better diagnosis and hemovigilance in Amazonas, focusing on peripheral areas, where financial resources are scarce and the people who are more exposed to infectious diseases and carcinogenic agents. Thus, we suggest further epidemiological and molecular studies in the municipalities of the interior in order to record previously unreported cases, to achieve better clinical guidelines, and to identify risk factors for leukemia development.
